# Detection and Quantification of Retinal Neovascularization Using BrdU Incorporation

**DOI:** 10.1167/tvst.9.9.4

**Published:** 2020-08-04

**Authors:** Yan Shao, Jianglei Chen, Xiao-Rong Li, Jian-Xing Ma

**Affiliations:** 1 Tianjin Medical University Eye Hospital, Eye Institute & School of Optometry and Ophthalmology, Tianjin, China; 2 Department of Physiology, The University of Oklahoma Health Sciences Center, Oklahoma City, OK, USA; 3 Harold Hamm Diabetes Center, The University of Oklahoma Health Sciences Center, Oklahoma City, OK, USA

**Keywords:** angiogenesis, retinal neovascularization, oxygen-induced retinopathy, BrdU

## Abstract

**Purpose:**

The retina is a commonly used model for angiogenesis research due to its special characteristics. Oxygen-induced retinopathy (OIR) provides a useful model to study ischemia-induced neovascularization (NV) and to develop anti-angiogenic therapeutics. The purpose of this study was to develop a simple, accurate, and less-subjective quantification method for retinal NV in the OIR model.

**Methods:**

To address this challenge, we combined the conventional vascular staining and BrdU labeling of newly formed vascular cells to detect and analyze retinal NV. With daily injections of BrdU, which was incorporated into the DNA of newly formed retinal vessels under the OIR condition, ischemia-induced retinal neovasculature with BrdU labeling was distinguished from pre-existing vasculature and accurately quantified using the ImageJ program.

**Results:**

Compared with conventional quantification methods using isolectin B4 staining of the entire vascular network, BrdU labeling allowed us to distinguish newly formed vessels from the pre-existing vessels and to objectively quantify the newly formed vessels, which was verified in OIR mice with intravitreal injections of an antibody-neutralizing vascular endothelial growth factor.

**Conclusions:**

BrdU labeling provides a useful and sensitive method for studying retinal NV and evaluating the therapeutic effects of medical interventions against pathological angiogenesis.

**Translational Relevance:**

Quantitative, straightforward, and objective observation and evaluation of pathologic neovasculature are important to study the pathogenesis of NV and therapeutic effects using animal models.

## Introduction

The retina is a thin layer of transparent tissue consisting of glial cells and neural cells that senses and converts the light to neural signals and transmits the signals to the brain for visual recognition.^1^ Anatomically, the retina lies outside the brain, but it originates from an outgrowth of the developing forebrain and is therefore considered a part of the central nervous system (CNS).^2^ Being the most accessible part of the CNS, the retina has become a popular model for studies of both physiological and pathological angiogenesis in the nervous system.^3^ There are several advantages to using the retina as a model to study angiogenesis. First, the transparency of the retina offers the convenience of detection. Second, the retina allows for two-dimensional observance of the vascular network. Finally, robust methods have been developed for whole-mount labeling of endothelial cells and interacting cell types that can be combined with high-resolution imaging.^4^

The retina is also a particularly well-suited model for studying vascular pathology. Oxygen-induced retinopathy (OIR), which was first developed in kittens,^5^^,^^6^ recapitulates hallmarks of retinal neovascularization (NV) in human conditions, including retinopathy of prematurity (ROP) and proliferative diabetic retinopathy (PDR). The model was then further perfected in mice^7^ and has become widely used and well established in the research field.^8^^,^^9^ However, an objective method for direct detection and quantification of neovasculature remains to be developed for retinal NV. There are mainly two categories of methods for quantification of retinal NV. One is a cross-sectional technique, which involves manually counting the pre-retinal vascular cells.^7^ This method requires multiple ocular sections and is labor intensive. The other is isolectin B4 staining of retinal vessels in the flat-mounted retina, which gives a full view of the retinal vasculature compared with the cross-sectional technique.^3^^,^^10^ The latter method is widely used for quantification of NV in the OIR model.^9^^,^^10^ However, a challenge of this method is the difficulty in distinguishing the ischemia-induced neovasculature from pre-existing retinal vasculature.

Retinal NV is usually defined as a disorganized formation of leaky, small-caliber vessels (i.e., pre-retinal tufts) located anterior to normal retinal vessels with frequent extension into the vitreous cavity.^11^ The criteria of NV are mainly based on the morphology of vessels; consequently, quantification of the NV relies on morphological changes and is subjective. Because it is challenging to distinguish neovasculature from pre-existing vasculature, a reliable, objective, and efficient method to quantify retinal NV in the OIR model remains an urgent need.

The thymidine analog 5-bromo-2-deoxyuridine (BrdU) can be incorporated into synthesized DNA, a technique that was first used to examine proliferation in the central nervous system.^12^ BrdU allows objective definition of replicating cells, and, more importantly, additional labeling can be performed with markers specific for ECs, such as lectin.^13^ With confocal microscopy, BrdU labeling allows phenotypic identification of proliferated cells and their stereological quantification.

Here, we combined conventional isolectin B4 vessel staining and specific BrdU incorporation into newly formed endothelial cells to develop a more objective, efficient, sensitive, and specific method to quantify retinal NV. Using this method, we closely monitored the new vessel formation starting from budding of small tufts to the pathological angiogenesis dominating most of the vascular network. With this highly specific staining of newly formed endothelial cells, we established an objective and straightforward strategy to quantify ischemia-induced NV in the retina of the OIR mouse. Furthermore, this technique provided a quantitative and sensitive method for evaluating the therapeutic effect of anti-angiogenic drugs on abnormal angiogenesis in animal models.

## Materials and Methods

All of the experimental procedures were approved by the Institutional Animal Care and Use Committee of The University of Oklahoma.

### Oxygen-Induced Retinopathy Mouse Model

The OIR model using newborn C57BL/6J mice was established as previously described.^7^ Briefly, mice at postnatal day 7 (P7) were exposed to 75% oxygen in an oxygen chamber until P12 (5 consecutive days). Starting at P11, mice received daily intraperitoneal injections of 100 mg/kg bromodeoxyuridine (BrdU, ab142567; Abcam, Cambridge, UK) until the day before euthanasia. At P12, pups were returned to room air. To evaluate the effects of an anti-vascular endothelial growth factor (VEGF) antibody on retinal NV, OIR mice received an intravitreal injection of 1 μg/eye (1 μg/μL) anti-VEGF antibody (AF-493-NA; R&D Systems, Inc., Minneapolis, MN) at P14. Littermates were injected with 1 μg non-specific murine immunoglobulin G (IgG) in phosphate-buffered saline (PBS) as controls.

### Vessel with BrdU Staining and Imaging

Eyes were enucleated and fixed in 4% paraformaldehyde for 24 hours. Retinas were isolated and incubated in 2-M HCL for 1 hour to hydrolyze DNA for BrdU staining. Retinas were permeablized in PBS containing 5% bovine serum albumin (BSA) and 0.3% Triton X-100 for 3 hours; they were then stained with 100 μL of a mixture of lectin (500 μg/mL; Isolectin B4), an antibody against BrdU (1:50, ab152095; Abcam), and 4′,6-diamidino-2-phenylindole overnight. Tyramide SuperBoost Kits with Alexa Fluor Tyramides (Thermo Fisher Scientific, Waltham, MA) were used to enhance the sensitivity of BrdU staining. The whole retinas were flat mounted, and images were captured at the same setting in each experiment using an Olympus Fluoview confocal microscope, Version 2.1a (10× and 60× objectives; Olympus Corporation, Tokyo, Japan) and converted to 16-bit images using Adobe Photoshop (Adobe, Inc., San Jose, CA).

### Quantification of NV Using BrdU Staining

ImageJ (National Institutes of Health, Bethesda, MD) was used to quantify retinal NV in OIR mice. Because BrdU staining (green color) labels the dividing vascular endothelial cells on the vascular network, the strategy of the quantification was to calculate the area percentage of BrdU (green channel) staining on the total vessel network (isolecin B4 staining, red channel). Briefly, an original merged (green and red) image taken by confocal microscope (10× objective) was opened in ImageJ. Individual channels were split and inverted into black-and-white images in ImageJ. The threshold was adjusted to mark the entire vessel network, and the selection was created (cyan color) based on it. The selection was applied on the green channel image, and the threshold was adjusted based on the BrdU staining and fixed in one set of the experiment. The percentage of green channel area fraction of the selection of the total vessel network was measured using ImageJ. The mask of the red channel was created, and the total area of the network was acquired by ImageJ measurement. Measurements of all petals of dissected retina were collected to calculate the neovascular percentage of the whole retinas.

### Statistical Analysis

Statistical analyses were performed using Prism 7.0 (GraphPad Software, San Diego, CA). Quantitative data are presented as mean ± *SEM* and were analyzed by Student's *t*-test when two groups were compared and by analysis of variance when more than two groups were compared. *P* < 0.05 was considered to be statistically significant.

## Results

### Visualization of Neovasculature and Total Vascular Network in the Retina

**Figure 1.**
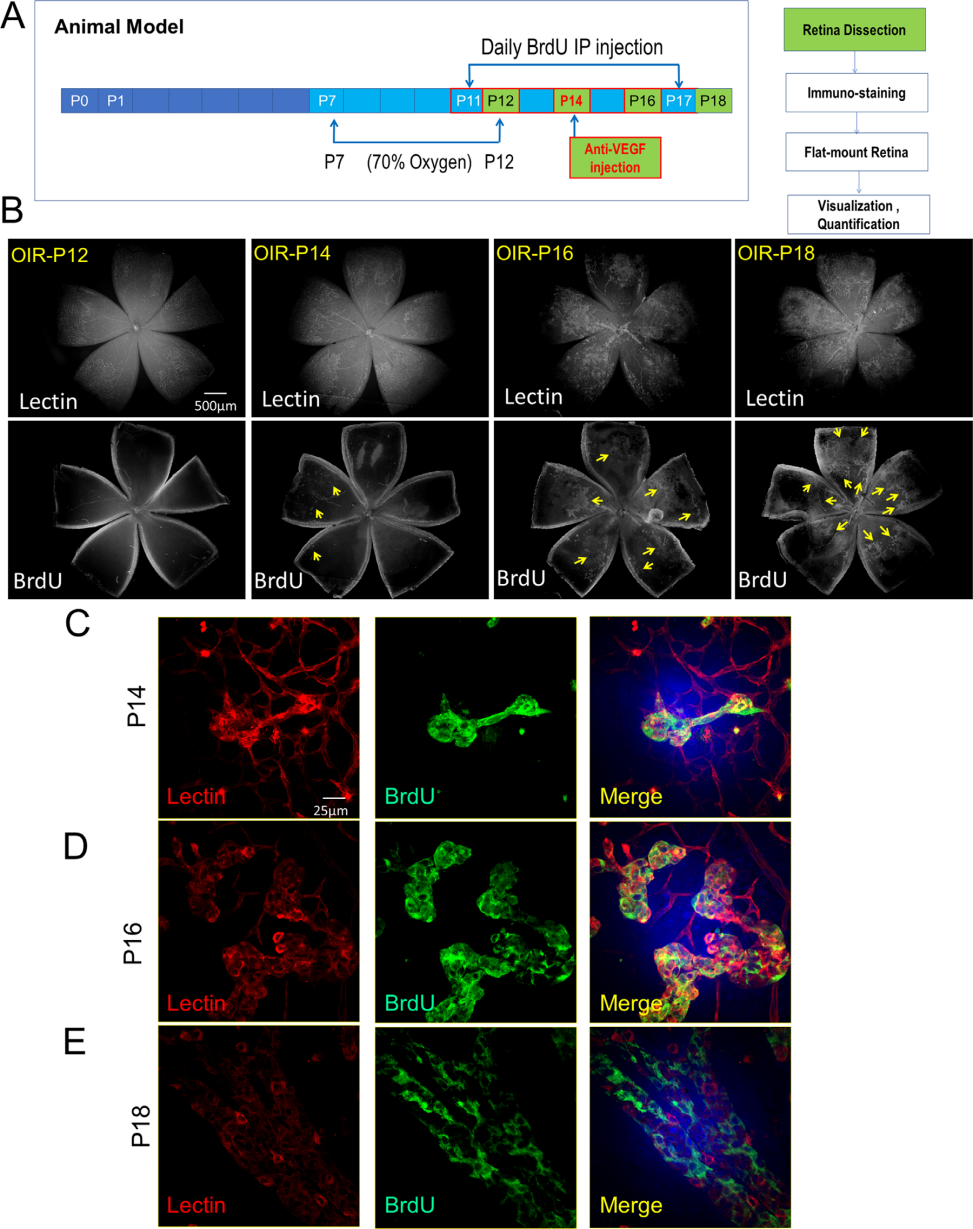
Detection of BrdU-labeled neovasculature in the OIR mouse retina. (a) The scheme of the OIR mouse model and experimental design. (b) Representative images of immunostaining of the flat-mounted retinas of OIR mice (P12, P14, P16, and P18). Isolectin B4 (lectin)-stained total retinal vascular networks and BrdU-labeled retinal neovasculature. Images were taken using an Olympus fluorescence microscope and converted to 16-bit images using Adobe Photoshop. The gray BrdU positive staining is indicated by *yellow arrows*. (c–e) High-magnification (60× objective) images of the retinal vascular tufts and neovasculature at different stages of OIR captured under a confocal microscope.

Utilizing isolectin staining of endothelial cells and BrdU labeling of newly formed endothelial cells, we developed a method to visualize and quantify NV in the retina of the OIR mouse (Fig. 1A). Isolectin staining allows us to view the entire vascular networks in flat-mounted retina. In contrast, BrdU labeling allowed us to specifically label only the neovascular cells through the BrdU fluorescence channel (Fig. 1B). In flat-mounted retina of OIR mice from P12 to P18, the BrdU fluorescence images showed increasing BrdU-positive cells in the vascular network (indicated by yellow arrows, Fig. 1B). As shown in the high magnification confocal microscope images (60×), the scattered neovascular tufts were clearly labeled by the BrdU staining in the retina of OIR mouse at the age of P14 (Fig. 1C). These “tufts” formed disorganized, dilated, and tortuous vessels resembling the pathological NV seen in human ROP or PDR.^14^ However, these budding tufts were very difficult to recognize in the conventional isolectin staining images (Fig. 1C). At a late stage, the accelerated growth of these tufts corresponding with vascular dilation and abnormal vascular growth was obvious in the BrdU staining (Fig. 1D). With the progression of retinal NV, the BrdU-positive vascular cells were detected in almost the entire peripheral retina (Figs. 1B, 1E). Compared with regular lectin staining showing the entire retinal vascular networks, the specific BrdU staining illustrated only the pathological neovasculature and distinguished it from pre-existing retinal vascular networks.

### Quantification of Neovasculature

**Figure 2.**
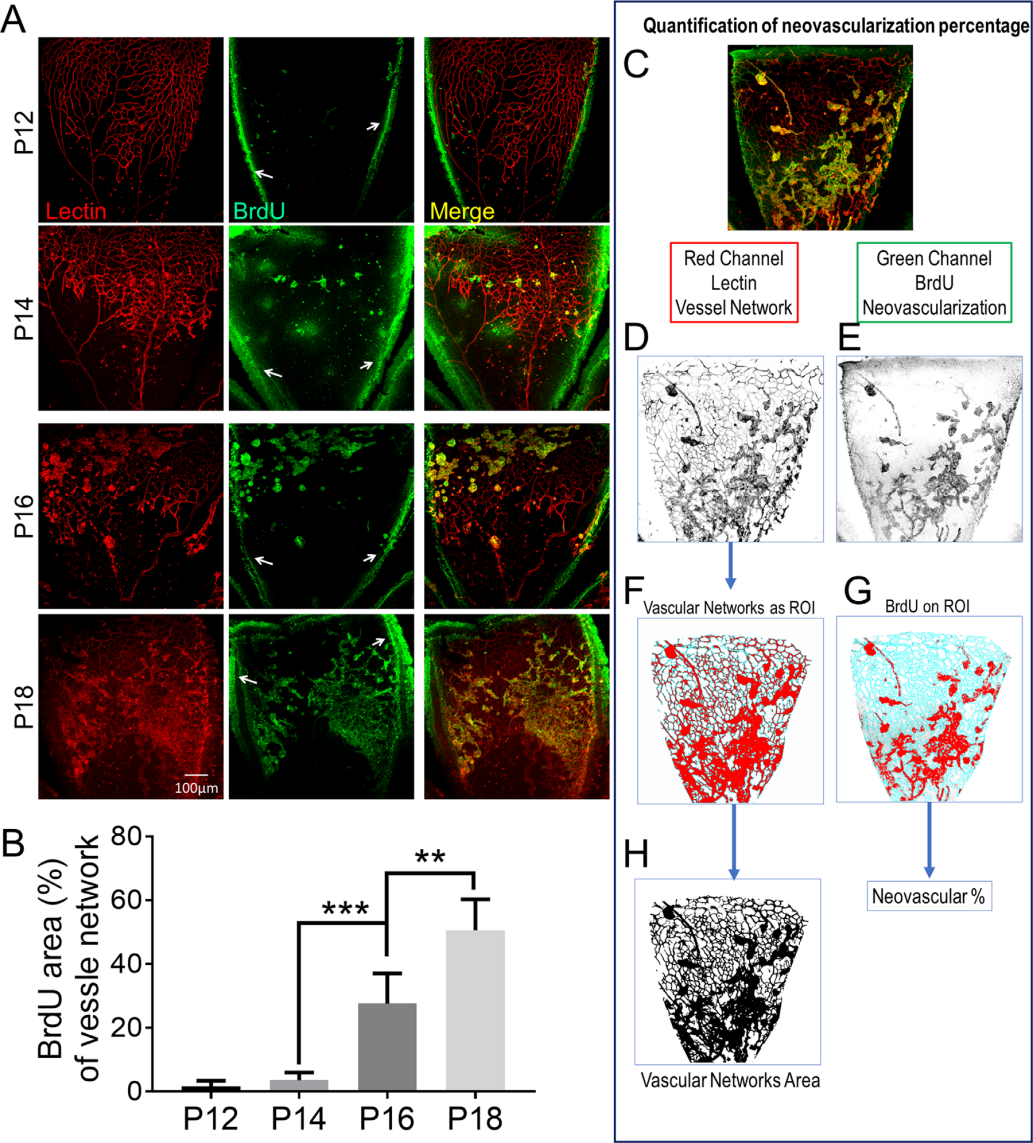
Quantification of NV in the retina of OIR mice using BrdU staining. (a) Lectin (*red*) and BrdU (*green*) immunostaining of one petal (five petals each retina) of the flat-mounted retina of the OIR mouse. The dissection edges of each petal are indicated by *white arrows*. (b) Quantification of BrdU staining (*green*) area as a percentage of the total vascular networks on each OIR mouse retina (mean ± *SEM*; *n* = 5; ^**^P < 0.01, ^***^P < 0.001), and the quantification method for the BrdU-positive neovasculature. (c) An original image taken under a confocal microscope (60×). (d) The inverted red channel using ImageJ. (e) The inverted green channel using ImageJ. (f) Regions of interest (ROI) created in ImageJ based on vascular network selection. (g) The green fluorescence channel overlapped the ROI created for vascular network selection. (h) The mask created in ImageJ to measure the area of the vascular networks.

Figure 2A shows representative confocal microscope images of flat-mounted retinas of OIR mice collected at P12, P14, P16, and P18. The progression of retinal NV was directly highlighted by the BrdU staining (green channel). Combining isolectin staining of total vascular networks (red) and BrdU labeling of newly developed vessels (green), the ratio of new vessel formation was quantified (Fig. 2B) using the basic ImageJ program following the strategy illustrated in Figures 2C to 2H. Noticeably, there was intense green fluorescence signal at the dissection edge of each petal. This non-specific fluorescence signal did not overlap with any vessels in the retina (indicated by the white arrows in Fig. 2A) and was excluded from the quantification (Figs. 2D–2H). The ratio of positive BrdU staining on the total vascular network demonstrated that the vessel growth was at a relatively slow pace before P14 but accelerated and reached almost 50% of the entire retinal vasculature at P18. This strategy provided a straightforward and relatively objective method to quantify the progression of angiogenesis in tissues such as retina and CNS.

### Evaluation of Therapeutic Effect of an Anti-VEGF Antibody

**Figure 3.**
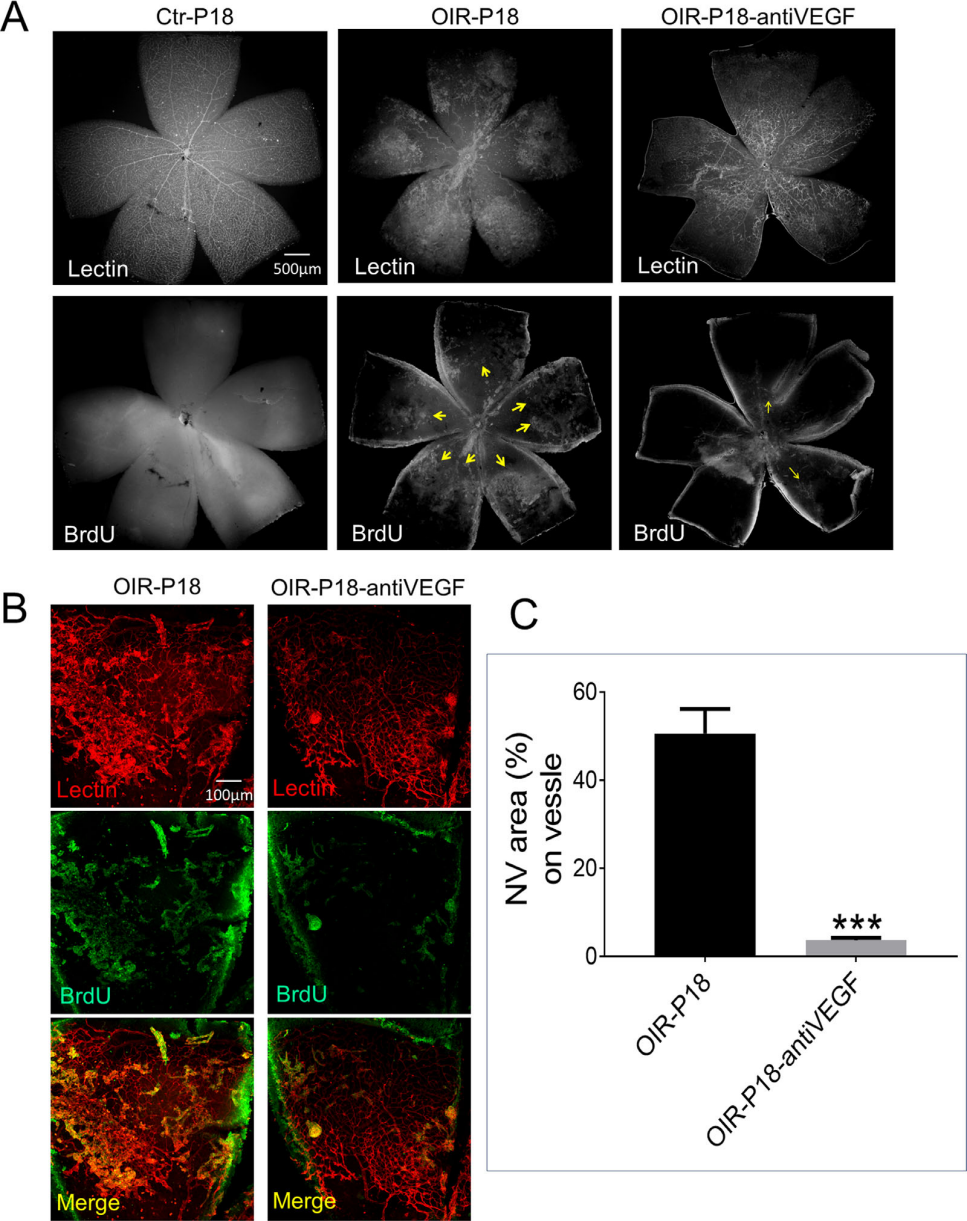
Evaluation of the effect of an anti-VEGF antibody on retinal NV in OIR mice. OIR mice received an intravitreal injection of the anti-VEGF antibody (1 μg/eye) at P14, with the same amount of non-specific murine IgG for control. (a) Representative images of immunochemistry staining of lectin and BrdU on the flat-mounted retina of P18 normoxic control (Ctr-P18), OIR mice injected with control IgG (OIR-P18), and OIR mice with the anti-VEGF antibody (OIR-P18-antiVEGF). The images were taken under a fluorescence microscope. The gray BrdU-positive staining is indicated by *yellow arrows*. (b) Confocal images of one petal of flat-mounted retinas of P18-Ctr, P18-OIR, and OIR-P18-antiVEGF mice stained with lectin and BrdU. (c) Quantification of BrdU-positive NV area as a percentage of total vascular networks (mean ± *SEM*; *n* = 5; ^***^P < 0.001).

To further demonstrate that this method was applicable to the evaluation of anti-angiogenic effects of therapeutics, a VEGF-neutralizing antibody was injected intravitreally into the OIR mice at P14, and its anti-angiogenic effect was examined using BrdU/isolectin staining. In the flat-mounted whole retina stained with isolectin and BrdU (Fig. 3A), we compared retinal NV in OIR mouse treated with the anti-VEGF antibody and OIR mice treated with non-specific IgG and with normoxic control. Compared with the normoxic control mouse retina, the OIR mouse showed an abnormal neovascular network and substantial BrdU staining in the retina. After injection of the anti-VEGF antibody, the BrdU staining was mostly abolished in the vascular networks of the retina, suggesting that the anti-VEGF antibody effectively inhibited the retinal NV in the OIR model. Using the quantification method mentioned above (Figs. 3B, 3C), we acquired the NV ratio indicating that anti-VEGF antibody almost completely prevented the pathological angiogenesis in the retina of OIR mice at P18.

## Discussion

Retinal NV is an important complication of multiple disorders. A simple and objective detection and quantification method for retinal NV is desired. Although the human retinal vasculature develops before birth, the mouse retinal vasculature develops after birth and therefore offers unique advantages for experimental manipulation.^15^

Ischemia-induced retinal NV in the OIR model develops a fast and dynamic NV process that provides a solid research model commonly used for the NV mechanism and anti-angiogenic therapeutic studies. Several advancements have been achieved toward establishing more rigorous and reliable methods to quantify the retinal NV in the OIR model. However, the traditional detection and quantification methods mainly depend on staining the entire vascular network and are laborious and subjective processes. The major challenge is to distinguish the neovasculature induced by ischemia from pre-existing physiological retinal vasculature. The present study innovatively combined BrdU DNA incorporation, which labels newly formed cells, with isolectin staining of endothelial cells. This method allows distinction of neovascular cells and thus provides an objective method to detect, monitor, and quantify the ischemia-induced new vessel formation in the retina of the OIR mouse. This method will provide a new tool for quantification of retina NV and evaluation of the therapeutic effects of anti-angiogenic drugs.

The current quantification methods of angiogenesis research using the OIR mouse model mainly depend on calculating the area of vaso-obliteration (VO)^10^ or tuft formation selected manually^10^ or by using computer software.^3^ However, the BrdU method in the current study allows not only viewing a “snapshot” of the total vessel network with isolectin staining at a time point but also monitoring the progression of NV.

Because the retinal NV is a dominant cell division process in postnatal retinal and major event in the OIR retina, BrdU staining is specific and sensitive to detecting new vessel generation. Based on previous studies of retinal development in the mouse, retinal cell division ceases by P11 in the neuroretina,^16^ especially the inner retina, which develops prior to birth.^15^ Hence, during ischemia-induced endothelial cell proliferation between P12 and P18 in the OIR model, the neuroretinal cells are quiescent and not labeled by BrdU. Our method takes advantage of this developmental trait in the mouse model, as BrdU is primarily incorporated into newly formed endothelial cells. As shown by *z*-stack function of a confocal microscope, the BrdU-positive cells were mainly observed on the inner surface of the retina and co-localized with retinal vessels, and the signal was quickly diminished toward the direction of the outer retinal. Most of the BrdU-positive cells were co-labeled with isolectin staining. All of these observations suggest that BrdU staining is a specific and unique tool for detecting quickly dividing vascular endothelial cells in the retina of the OIR mouse.

The BrdU method also provides a more direct and reliable approach to evaluate the effects of anti-angiogenic therapeutics using the OIR model. The development of neovasculature is very robust in OIR mice; however, the NV variation is wide in this model, which complicates the evaluation of efficacies for anti-angiogenic drugs. On the other hand, one intrinsic limitation of the conventional threshold-dependent NV quantification method is that it is challenging to distinguish the pathological NV from pre-existing vessels after therapeutic intervention. The specificity of BrdU labeling together with isolectin staining sufficiently overcame these pitfalls, as only the newly developed vessels under ischemia could be labeled by BrdU and observed and objectively quantified to evaluate efficacy of medical interventions.

Overall, this BrdU method for studying angiogenesis offers the following advantages:

1. It is less labor intensive and highly efficient compared to quantification of pre-retinal vascular cells on multiple cross sections.
2. It is more objective in the quantification of NV compared to traditional measurement of NV area in flat-mounted retina.
3. It distinguishes between vasodilation and angiogenesis. The initial stage of angiogenesis is the dilation of existing vessels, and the following stage is the endothelial cell proliferation and migration to form lumens.^8^ BrdU is incorporated into the DNA of dividing cells, which distinguishes the proliferative ECs from vasodilation.
4. It identifies the NV initiation process, including sprouting sites or tuft merging and development.
5. It dynamically monitors NV progression.

The limitation of our methods is that the mice must be euthanized to allow for retina isolation and staining; thus, methods for in vivo observation and quantification of NV are still eagerly sought in the research field.
